# Gut microbiota profiles of commercial laying hens infected with tumorigenic viruses

**DOI:** 10.1186/s12917-020-02430-3

**Published:** 2020-06-29

**Authors:** Xianhua Wan, Laipeng Xu, Xiangli Sun, Hui Li, Fengbin Yan, Ruili Han, Hong Li, Zhuanjian Li, Yadong Tian, Xiaojun Liu, Xiangtao Kang, Zhenya Wang, Yanbin Wang

**Affiliations:** 1 College of Animal Science and Veterinary Medicine, Henan Agricultural University, Zhengzhou, 450000 China; 2 College of Environmental and Resource Sciences, Henan Agricultural University, Zhengzhou, 450000 China; 3 Henan Research Center of Germplasm Resources for Poultry, Zhengzhou, 450002 China; 4 Key Laboratory of “Runliang” Antiviral Medicines Research and Development, Institute of Drug Discovery & Development, Zhengzhou University, Zhengzhou, 450001 China

**Keywords:** ALV-J, MDV, REV, Fecal microflora, 16S rRNA

## Abstract

**Background:**

Studies have shown that some viral infections cause structural changes in the intestinal microflora, but little is known about the effects of tumorigenic viral infection on the intestinal microflora of chickens.

**Results:**

A 29-week commercial layer flock positive for avian leukosis virus-J (ALV-J), Marek’s disease virus (MDV) and avian reticuloendotheliosis virus (REV) was selected, and fresh fecal samples were collected and examined for the composition of the gut microflora by Illumina sequencing of the V3-V4 region of the 16S rRNA gene. The operational taxonomic units (OTUs) of the fecal microbiota differentiated the chickens infected with only ALV-J and those coinfected with ALV-J and MDV or REV from infection-negative chickens. The enrichment and diversity of cloacal microflora in chickens infected with ALV-J alone were slightly different from those in the infection-negative chickens. However, the diversity of cloacal microflora was significantly increased in chickens coinfected with both ALV-J and MDV or REV.

**Conclusions:**

The intestinal microbiota was more strongly disturbed in chickens after coinfection with ALV-J and MDV or REV than after infection with ALV-J alone, and there may be underlying mechanisms by which the capacity for the stabilization of the intestinal flora was impaired due to viral infection and tumorigenesis.

## Background

Avian leukosis virus-J (ALV-J), Marek’s disease (MD) virus (MDV) and avian reticuloendotheliosis virus (REV) are tumorigenic viruses that cause immunosuppressive, oncogenic and runting syndrome in layer chickens [1–3]. There are currently no commercial vaccines or effective drugs for controlling ALV-J and REV infections, resulting in great economic losses in the laying hen industry [4].

ALV-J is an avian retrovirus that was first isolated from meat-type chickens and mainly induces myelocytomatosis, nephromas and immunosuppression [5]. The prevalence of ALV-J, one of the major diseases in commercial layer flocks, has caused this dangerous disease to become a serious threat to the poultry industry worldwide, especially to local breeds in China in recent years [6–11]. Myeloid leukosis, hemangiomas and leiomyosarcomas are the main clinical manifestations of ALV-J that are observed simultaneously in commercial layer flocks with ALV-J infection [12, 13]. REV is an avian retrovirus that causes lymphocyte tumorigenesis, immunosuppression, growth retardation and runting syndrome in chickens and turkeys [4]. REV may be transmitted horizontally by direct contact between birds and indirectly by mosquitos and contaminated vaccines against fowlpox [14–16], Gallid herpesvirus 2 [17, 18] and MD [19]. Serologic studies have confirmed that reticuloendotheliosis viral infection is common in commercial layer, broiler, and turkey flocks worldwide [20–22]. The results of a serological survey in China from 2005 to 2015 showed that the virus-positive rate increased from 7 to 15%, indicating that the virus has become common in commercial chicken flocks. Coinfection with ALV-J and REV has more severe consequences for growth, immunosuppression and mortality than infection with ALV-J or REV alone [23–25]. MD is a lymphoproliferative disease of chickens caused by an avian herpes virus, MDV. Currently, although there are vaccines available to prevent this disease, there is a clinically frequent occurrence of MD that may be due to the common occurrence of ALV-J infection, which leads to immunosuppression against vaccines [26]. The virus targets lymphoid tissue, such as the bursa of Fabricius, liver, and spleen, where tumors eventually develop, resulting in high mortality.

The interplay between microbial communities and hosts and interactions between microbes are essential for gut homeostasis [27, 28]. A balanced commensal gut microbiota is important to ensuring the health of the host [29]. The relationship between intestinal bacteria and host health has received widespread attention [30–32]. Gut microbes lower the pH through metabolites, participate in carbohydrate metabolism and lipid metabolism through metabolic enzymes, provide nutrients to the host by synthesizing amino acids and vitamins, and nourish intestinal epithelial cells to strengthen the gut barrier by producing short-chain fatty acids [33]. Gut microbial communities are influenced by diet, gender, age, and breed in a dynamic stable state of equilibrium [34–36]. The equilibrium is easily broken upon attack by various pathogenic factors, which may lead to disease and reduced production performance. Many studies now report intestinal microbial changes after infection with a wide range of pathogens, including bacteria, fungi and their toxins, parasites and viruses, which results in a decrease in the abundance and diversity of the intestinal flora [37–42].

Yitbarek et al. reported that influenza viral infection in chickens results in a shift in the gut microbiota and disrupts host-microbial homeostasis. The use of probiotic- and/or fecal microbiota transplantation (FMT)-based interventions can promote chicken recovery from H9N2 infection [29]. Li et al. reported that infectious bursal disease virus (IBDV) is able to replicate in gut-associated lymphoid tissues (bursa, cecal tonsils and cecum), inducing histological lesions, strong local immune cell changes and alteration of the gut microbiota composition, which led to increased susceptibility to pathogens invading the gut [42]. Perumbakkam et al. also reported that MDV infection is able to induce changes in the core gut microbiome of chickens after the early and late cytolytic phases of viral replication [43]. Related to avian leucosis viruses (ALV), the cecal microbiome has been shown to differ significantly following infection of specific pathogen-free (SPF) chickens with viruses from the subgroups J or K [38]. However, little is known about the additive effects on the intestinal microflora in adult chickens and on the resulting immune status that may arise from coinfection in the same animal with more than one avian tumorigenic virus, which likely have consequences on global health and susceptibility to other diseases.

Some studies have shown that dual infection and triple infection with ALV-J and other viruses such as MDV are common [44] and that these multiple viral infections result in enhanced pathogenicity [45] and a high level of tumor appearance. Multiple infections with tumorigenic viruses are largely responsible for tumorigenesis [10]. It must be noted that tumorigenesis caused by ALV-J infection in poultry is a chronic process and a true multifactorial disease that has proven very difficult to reproduce experimentally in SPF birds [46].

Taking all these observations into account, we decided in the present study to select a Chinese commercial poultry farm flock where ALV-J outbreaks have been identified and deaths have been recorded in chickens with different degrees of tumor development associated with infection with MDV or REV. Then, layer hens were selected from this flock as being infected with ALV-J alone or coinfected with ALV-J and MDV or REV, and the microbial composition of feces was analyzed using high-throughput sequencing technology. The aim is to explore the effects of different avian tumorigenic viral infections on the chicken intestinal microbiota. The expectation is to lay the foundations to further explore the mechanisms underlying the interaction between the intestinal microbiota and avian tumorigenic viruses and the consequences on the health status.

## Results

### Analysis of intestinal flora composition

The intestinal microbial communities from three groups of 18 samples were identified at different levels.

**Fig. 1:**
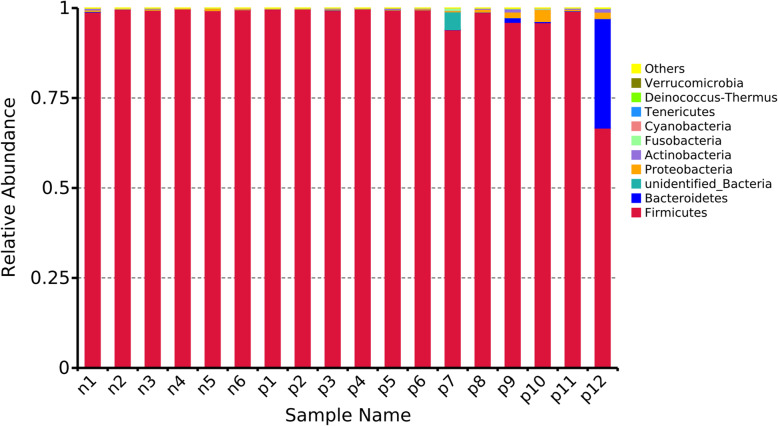
Relative abundances in bacterial communities at the phylum level. The abscissa shows the sample name; the ordinate represents the relative abundance; and “Others” represents the sum of the relative abundances of all the taxa except taxa shown in the figure

In fecal samples from the chickens of the N group, the preponderant bacteria at the phylum level were Firmicutes (99.36%). The main bacterial phyla of the chicken fecal infected with ALV-J were Firmicutes (99.55%) and Proteobacteria (0.20%). For the chickens infected with ALV-J and MDV or REV, the main bacterial phyla were Firmicutes (91.79%) and Bacteroidetes (5.38%) (Fig. 1).

**Fig. 2:**
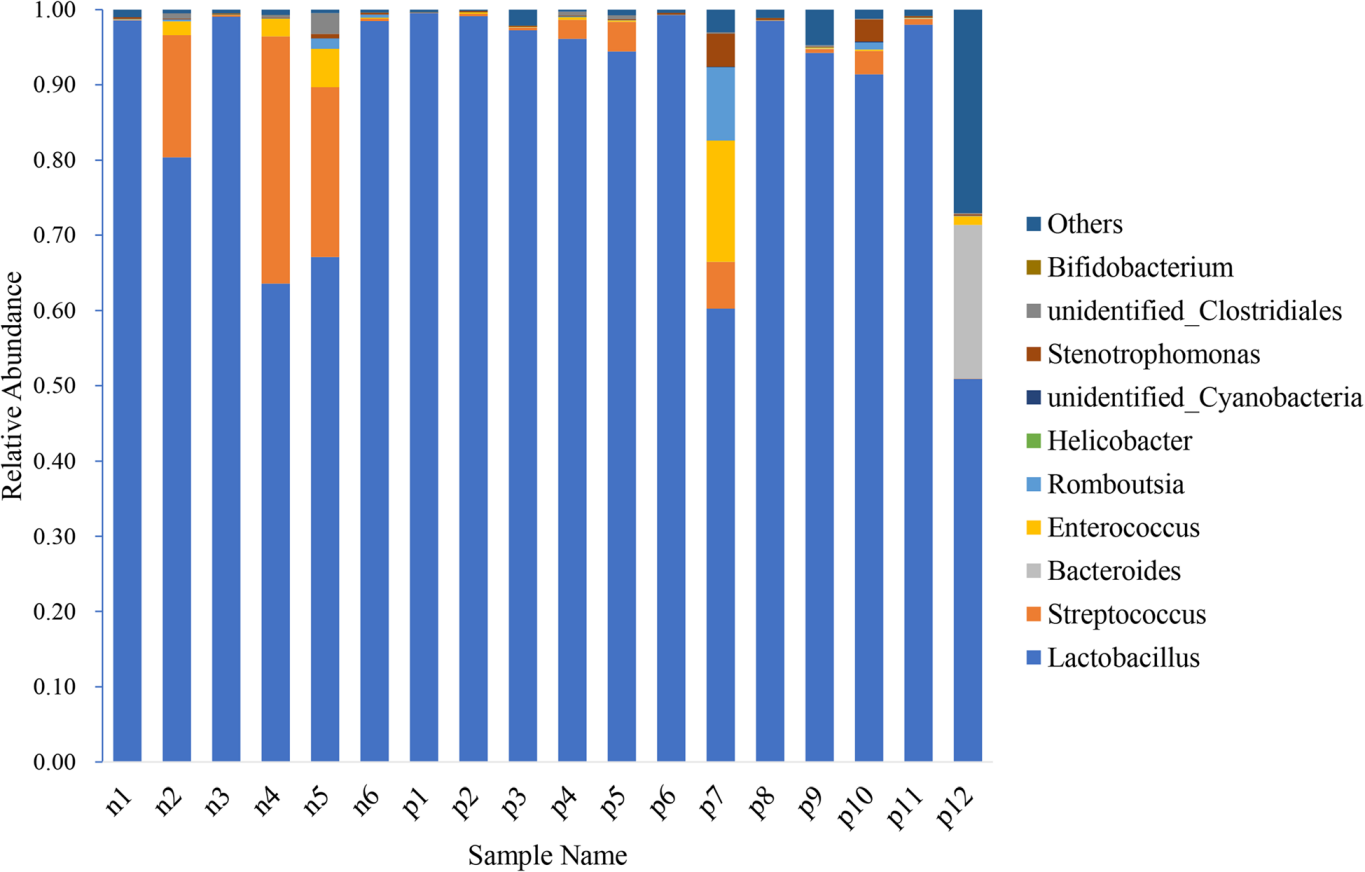
Relative abundances in bacterial communities at the genus level. The abscissa shows the sample name; the ordinate represents the relative abundance; and “Others” represents the sum of the relative abundances of all the taxa except taxa shown in the figure

At the genus level, the main intestinal bacteria of the N group were Lactobacillus (84.52%), Streptococcus (12.03%) and Enterococcus (1.58%). The main intestinal bacteria of the chicken P1 group were Lactobacillus (97.61%), Streptococcus (1.18%) and Enterococcus (0.15%). Meanwhile, Lactobacillus (82.20%), Bacteroides (3.43%) and Enterococcus (2.94%) were predominant in the chickens of the P2 group (Fig. 2).

Comparisons of intestinal bacterial communities at the phylum and genus levels among the three groups were performed. The results showed that there was no significant difference in the intestinal bacterial community between the three groups at the phylum and genus levels (*P* > 0.05).

**Fig. 3:**
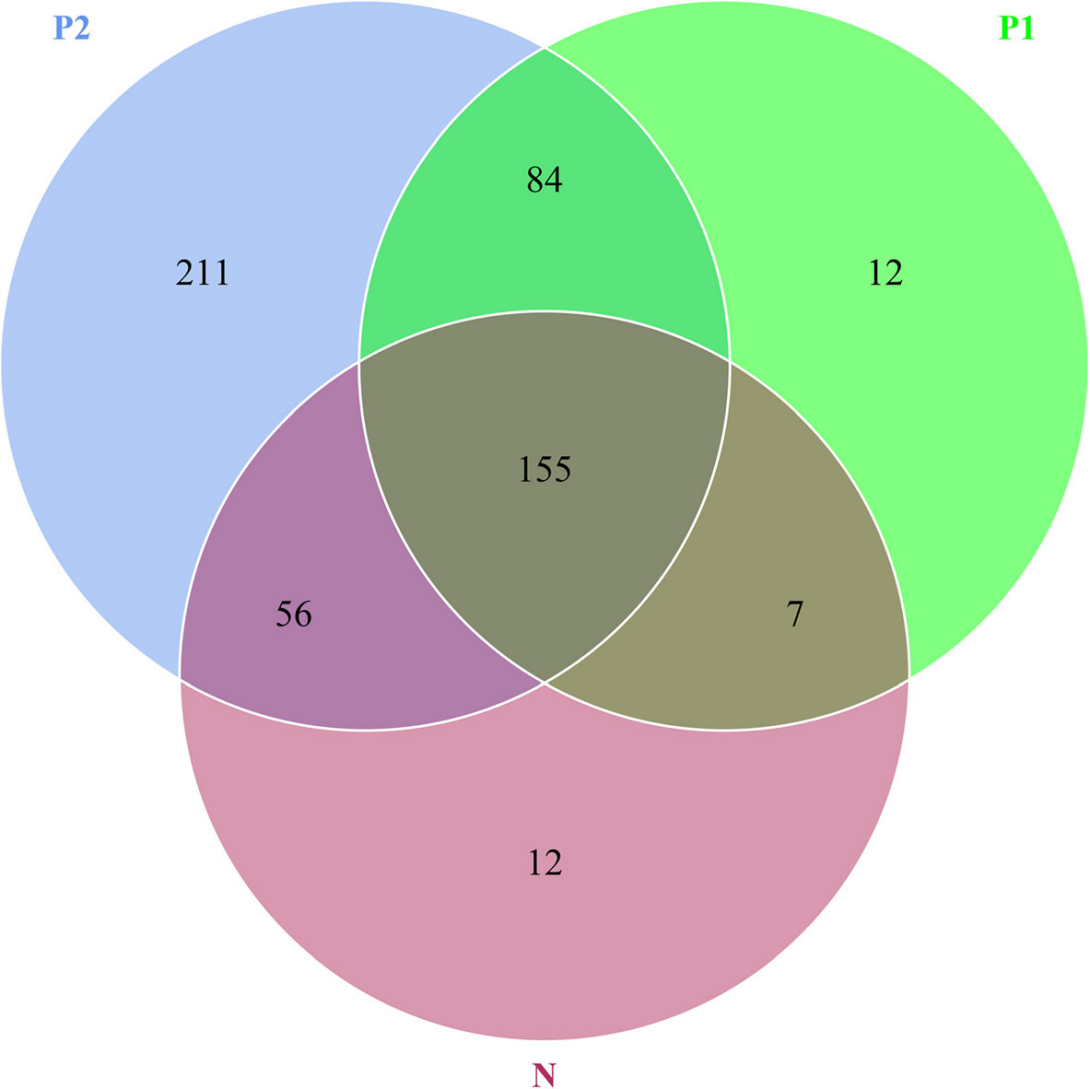
Venn diagram of the intestinal flora structure. Each circle in the figure represents a sample, and the numbers in overlapping circles show the overlap between representative samples. The numbers of OTUs in nonoverlapping circles represent the numbers of unique OTUs in the samples

Further analysis of the effective tags of groups N, P1, and P2 (230, 258 and 506 OTUs, respectively) showed that 155 OTUs were shared among all groups. Groups N and P1 shared 162 OTUs, groups N and P2 shared 211 OTUs, and groups P1 and P2 shared 239 OTUs. In addition, 12 OTUs were unique to group N, 12 OTUs were unique to group P1, and 211 OTUs were unique to group P2 (Fig. 3).

### Alpha-diversity index

The microbial complexities in the gut of chickens were estimated on the basis of alpha-diversity indices (Chao1 indices and Shannon indices). Chao1 was used to estimate species richness, while Shannon’s index was used to indicate species diversity. The results showed that P2 samples had the largest alpha-diversity indices, followed by P1 and N (Table 1).

**Table 1:** Alpha-diversity indices from samples

Sample name	Chao1	ACE	Simpson	Shannon
^n^1	174.25	185.867	0.65	2.314
^n^2	115.4	123.013	0.796	2.837
^n^3	111.65	121.056	0.809	2.816
^n^4	103.053	110.75	0.78	2.79
^n^5	111.65	120.15	0.846	3.253
^n^6	112.5	116.492	0.586	2.105
^p^1	121.067	118.74	0.693	2.281
^p^2	100.5	106.726	0.648	2.012
^p^3	181.812	195.909	0.767	2.746
^p^4	103.048	112.166	0.787	2.738
^p^5	120.6	128.981	0.791	2.825
^p^6	96.455	101.994	0.709	2.569
^p^7	153.84	156.115	0.888	3.656
^p^8	166.533	186.451	0.775	2.732
^p^9	273.5	277.218	0.832	3.262
^p^10	190.333	188.509	0.733	2.763
^p^11	77.929	80.859	0.704	2.32
^p^12	359.111	360.692	0.936	5.317

### Principal coordinate analysis

**Fig. 4:**
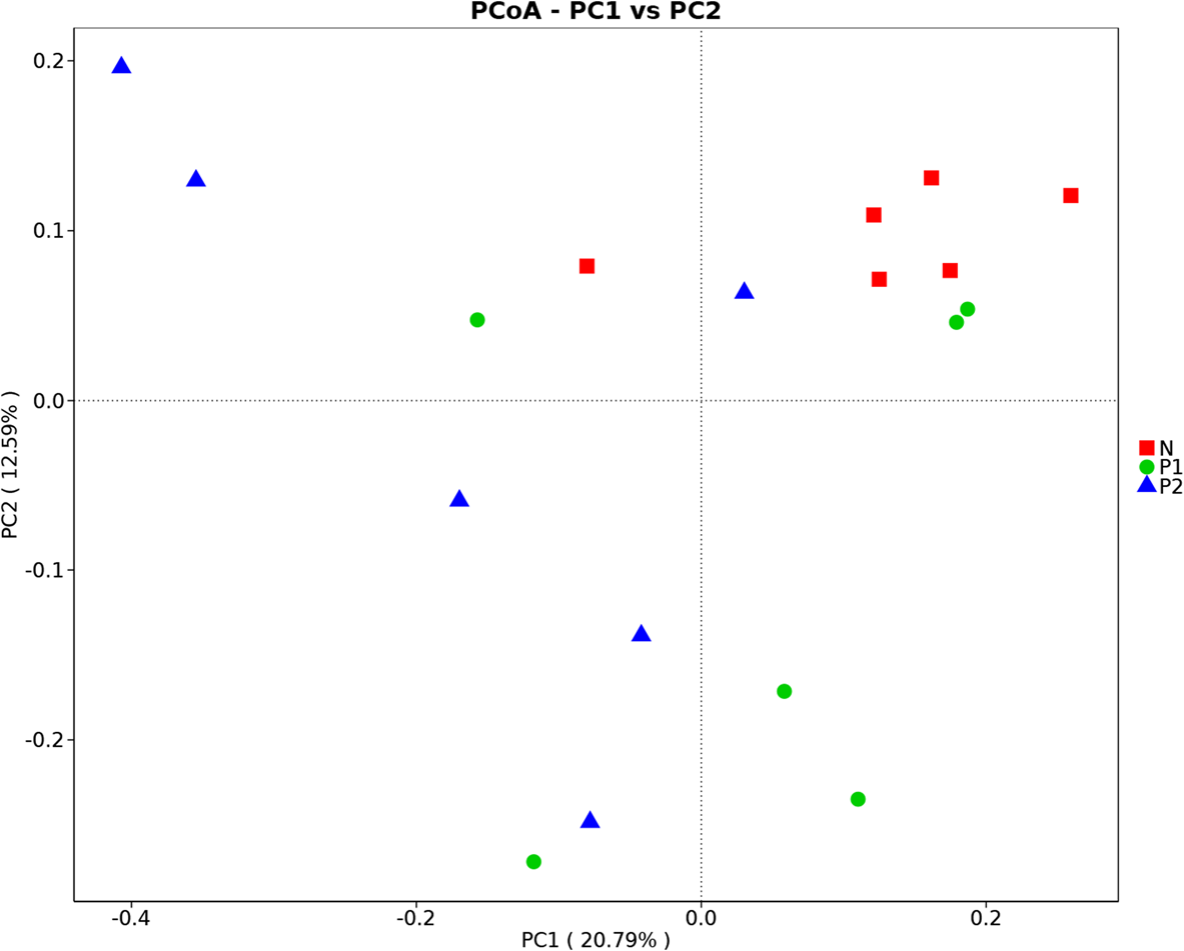
Principal coordinate analysis. The abscissa and ordinate each represent a principal component, and the percentage represents the amount of variation between samples explained by the principal component; each point in the figure represents a sample, and the samples in the same group are shown in the same color

A PCoA identifies the most important elements, and it extracts structure from multidimensional data through eigenvalue and eigenvector ordering. It can intuitively show whether the sample community structure of each group is different by the distance between the samples. As shown in Fig. 4, the distances between the 6 samples in group N were relatively small, indicating that the community difference was small; samples in groups P1 and P2 were relatively scattered, and the distances between samples were relatively large, indicating a large difference in communities in samples P1 and P2.

### Principal component analysis

**Fig. 5:**
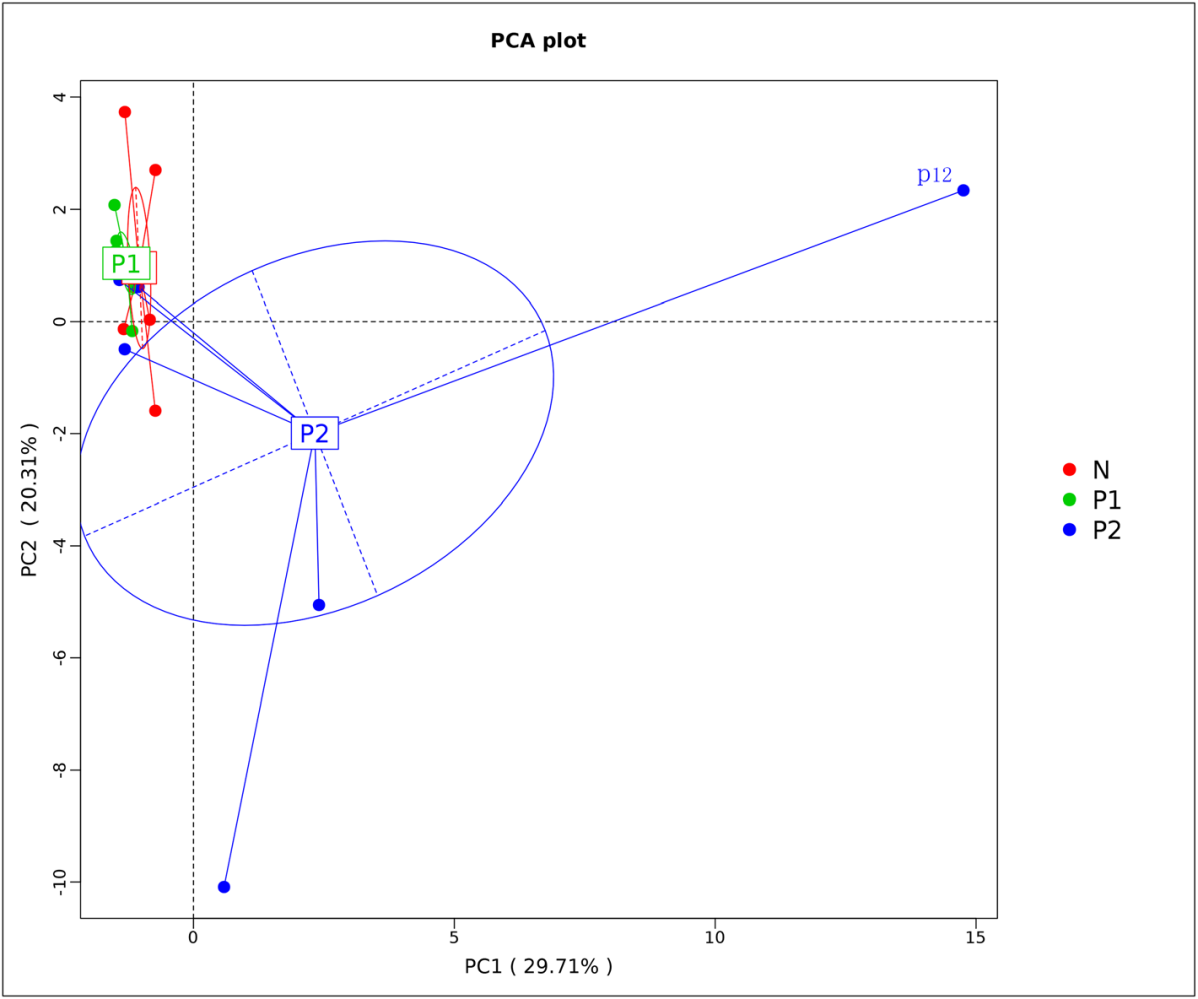
Principal component analysis diagram. Samples with more similar flora structures are closer in distance; otherwise, the distance between samples is greater

PCA revealed that infection with ALV-J and MDV or REV at the same time altered the bacterial communities in the gut of chickens when compared to uninfected chickens and chickens infected with only ALV-J (Fig. 5). Clear differences were observed in the bacterial communities between the N and P2 groups. However, no clear differences were observed in the community composition of bacteria in the gut samples between chickens infected with only ALV-J and uninfected chickens. Overall, the intestinal microbiota was more strongly disturbed in chickens after coinfection with ALV-J and MDV or REV than after infection with ALV-J alone.

### Cluster heatmap of the relative abundances of species

**Fig. 6:**
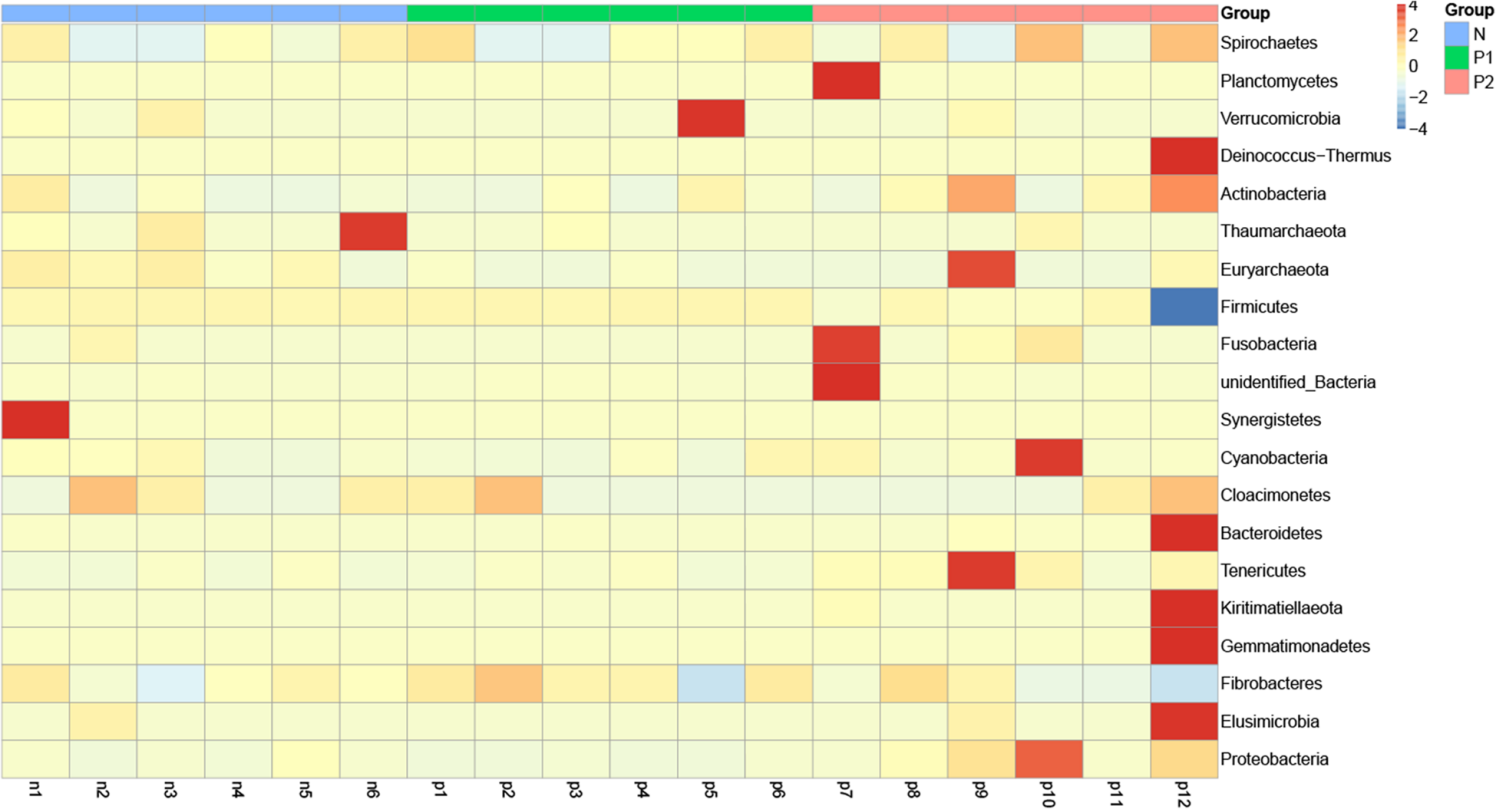
Cluster heatmap of relative species abundance. The vertical axis provides the sample information, and the horizontal axis provides the species annotation information. Red represents the phyla with a high abundance in the corresponding samples, while blue represents phyla with a low abundance

A heatmap of the 20 most abundant phyla was constructed for comparative analysis (Fig. 6). The composition of the intestinal microbiota showed obvious similarity based on ALV-J infection and healthy chickens, but for individuals, n1, n6 and p5 showed greater differences. However, in the samples infected with ALV-J and MDV or REV, there were abundant and obvious differences in multiple phyla between samples. The stable phylum in the first two groups also changed to varying degrees, and the phylum of the p_12_ sample was the most obviously changed. Sample p12 presented increases in the phyla Deinococcus-Thermus, Actinobacteria, Bacteroidetes, Gemmatimonadetes and Elusimicrobia, while the phylum Firmicutes decreased obviously.

### Column chart of the relative abundances of functional annotations

**Fig. 7:**
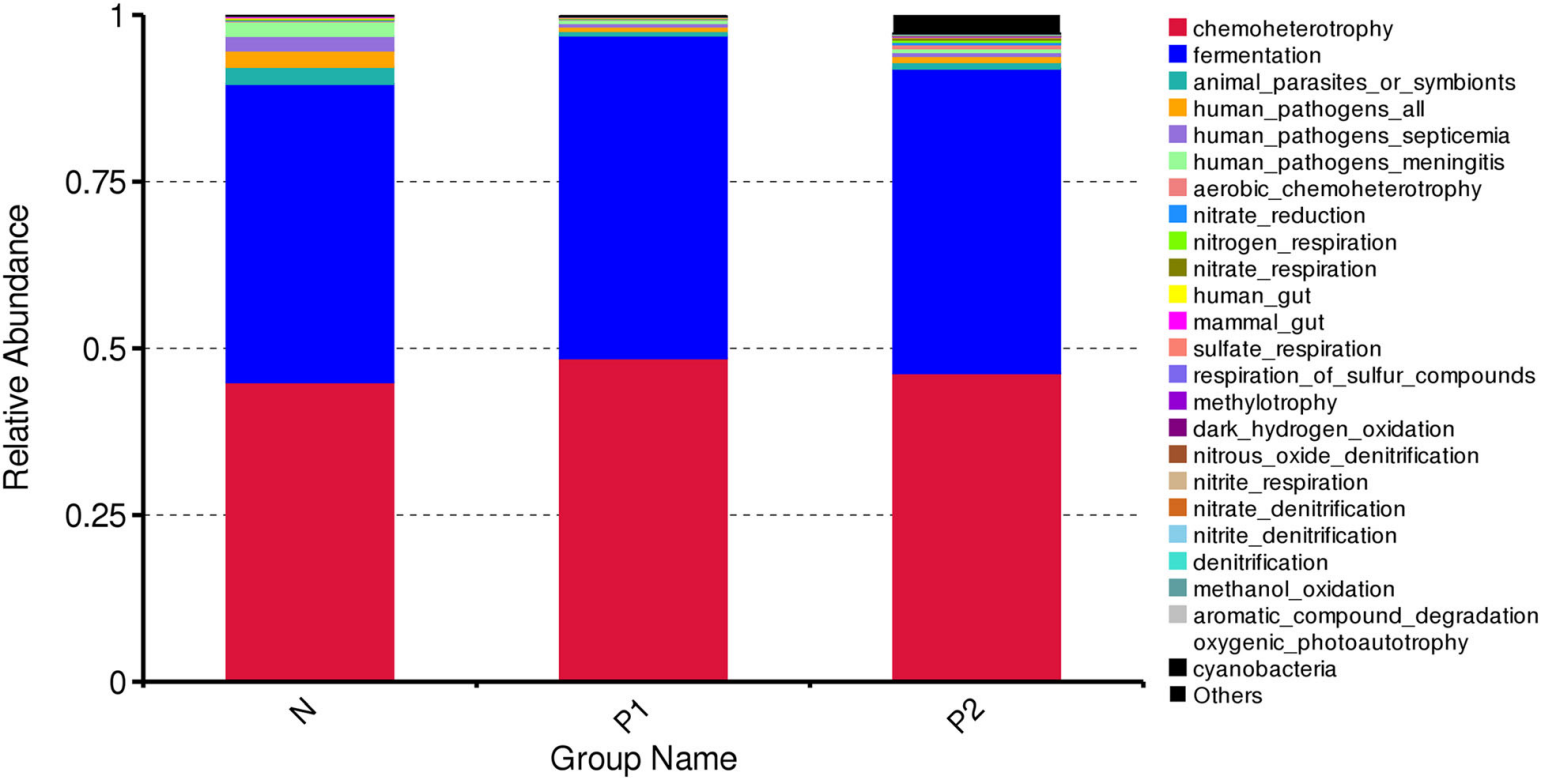
Column chart of the relative abundances of the Tax4Fun functional annotations**.** Different colors represent different functional annotations

To investigate the influence of tumorigenic viral infection on the functional performance of the microbiota, we performed Tax4Fun analysis of the three groups. As shown by the column chart of functional annotations based on the abundance of functional information in the annotation hierarchy (Fig. 7), we compared predicted microbial functions among three groups and detected that chemoheterotrophy and fermentation were most enriched. Although viral infections did not cause significant changes in these two main functions of the gut, other functions were affected to a certain extent. Chickens infected with ALV-J and MDV or REV had more intestinal microbial dysfunction than those infected with only ALV-J.

## Discussion

Comparing gut microbiota composition in layer hens from a commercial flock shown to be infected by three different tumor-inducing avian viruses (ALV-J, MDV and REV), we established that the gut microbiota balance was disturbed differentially according to the infection by one (ALV-J, P1 group) or several (ALV-J and REV or MDV, P2 group) viruses compared to that of the control group (uninfected and apparently healthy, N group). The number of OTUs in the P2 group was much larger than those in the N and P1 groups. Moreover, the diversity of the flora was increased in the case of coinfection compared with infection with ALV-J alone. Our results corroborate the existing literature on intestinal microbiota changes following infection with avian tumor-inducing viruses. We further demonstrated that simultaneous infections by different viruses result in more severe disturbances of the gut microbiota composition [38, 43, 47]. The PCoA results showed a large difference in the fecal microbiota communities of tumorigenic virus-infected chickens, suggesting that the fecal microbiota compositions were changed by tumorigenic viral infection. The PCA displayed consistent results with the PCoA in classifying the fecal samples. In addition, the PCA results showed that the fecal microbiota compositions of dually infected individuals varied more than those of the ALV-J-infected individuals.

This pattern of intestinal flora disorders associated with infection with ALV-J alone or coinfection with different avian tumor-inducing viruses might be supported by the appearance of physical/functional digestive disorders induced by these viruses and the alteration of the dialog between the host and the gut microbiota through immunosuppression [38, 43, 47]. The changes in the intestinal microbiota balance may favor infection with pathogens from the digestive tract and negatively affect chicken health. Intestinal epithelial cells infected by various pathogens easily lose their integrity, enabling microbes and metabolites to translocate and invade mucosal vessels and thus cause systemic infection and septicemia [47, 48]. This means that gut intestinal changes after infection by ALV-J may favor the infection/replication of MDV and/or REV and tumor development, thus exacerbating the disease and its consequences.

Specific changes that may be observed in the gut microbiota composition after tumor-inducing infection have been observed in previous studies. The gut microflora from ALV-J predominantly includes opportunistic pathogens in Firmicutes, such as members of *Staphylococcu*s and *Weissella*, and some genera of *Bacillales*. Moreover, [38] opportunistic pathogens such as *Escherichia-Shigella* and *Enterococcus*, members of Erysipelotrichaceae in Firmicutes and members of Helicobacteraceae in Bacteroidetes increase sharply in ALV-J-infected chickens. We hypothesized that the effect of viral infection on the intestinal microbial community mainly depends on the tissue tropism of the virus for target organs [49]. Studies on ALV-J-induced pathology have shown that the virus induces the formation of several types of tumors, such as hemangioma, leiomyosarcoma and myeloma (immune system). Thus, similar to REV and MDV, ALV-J most likely induced immunosuppression as the tumors progressed [11, 50]. The clinical course of RE and MD neoplastic diseases targeting the immune system may be acute and chronic, as for ALVJ-induced disease. The viruses cause immunosuppression through depletion of lymphocytes for MDV and REV and tumorigenesis affecting T lymphocytes for MDV and B lymphocytes for REV in natural conditions [1]. Accordingly, this broad immunosuppression may strongly impact the gut immune system-microbiota balance. Moreover, solid lymphoid tumors may localize in different organs, including mostly lymphoid tissues (bursa, spleen) but also frequently the liver. In addition, lesions of the digestive tract, such as proventriculus lesions, enteritis and liver necrosis may be observed, especially for REV [49], which probably disturbs gastrointestinal functioning. Thus, it may also be hypothesized that this is another important cause of gut microbiota imbalance. In conclusion, we strongly suppose that simultaneous infection by more than one tumor-inducing virus in chickens has an additive effect on the immunosuppressed status and the alteration of gut function and integrity, leading to disruption of the dialog between the microbiota and the host, and to increased microbiota disturbances, as we observed [4, 24, 51].

In addition to an increase in the abundance of opportunistic bacteria, a large number of microorganisms that normally do not reside in the gut or reside at very low levels such as *Cyanobacteria* and *Actinobacteria* were found to increase in chickens with double infection by ALV-J and MDV or REV [52]. These results are consistent with those of a report [53] showing that the diversity of the cecal microflora increased in chickens infected with ALV-J. By contrast, when referring to other pathogens inducing serious damage to intestinal tissues and causing enteritis and diarrhea, such as Eimeria tenella, a sharp decrease of the diversity of the cecal microflora [53]. Thus, infection with several tumor-inducing viruses that are strongly deleterious to the immune system may differently impact the dialog between the host and the microbiota at the gut level, resulting in increasing bacterial diversity, although being able to alter somehow the gut integrity.

Chickens infected chronically with ALV-J exhibit most of the time normal feed and water intake, and no interruption of egg laying [6, 8]. By contrast, increased alteration of normal gut function that may follow coinfection with several tumor-inducing viruses, as previously described, may result in alteration of feed intake and decreases in nutrients favorable to gut microbiota richness. There were differences in the functional annotation of the intestinal microorganisms between chickens infected with ALV-J alone and chickens with coinfections of ALV-J and MDV or REV. This difference is probably due to the weak intestinal microbiota stability and vulnerability to exogenous bacteria, leading to functional disorders. Therefore, mixed infections are more likely to cause substantial changes in the number and species of flora than are single ALV-J infections and lead to structural disorders in the chicken flora.

## Conclusion

This study is the first to compare the effects of infection with ALV-J and coinfection with other tumor-inducing viruses, MDV and REV, on layer hens, as commonly observed in the field. We confirmed that infection with ALV-J can lead to gut microbiota structural changes. The main manifestations were a decrease in the phylum Firmicutes and an increase in the phyla Bacteroidetes and Proteobacteria and unidentified bacteria. We show that coinfection increased the diversity of unclassified bacteria and Cyanobacteria and Actinobacteria but reduced the richness of the dominant members of the flora usually present in noninfected chickens, despite none of the chickens displaying any symptoms. This let us suppose an alteration of the dialog between the host and the gut microbiota, presumably due to additive effects of these viruses through persistent immunosuppression and increased gut function alteration and lesions. Further studies are needed to understand how the improvement of the gut microbiota imbalance by appropriate diets and/or probiotics may be beneficial to resistance to avian chronic neoplastic diseases.

## Methods

### Experimental design

Eighteen 29-week-old commercial laying hens were selected according to their different infections, of which six healthy chickens formed the negative control group and were numbered n1-n6; six chickens infected with ALV-J formed group P1 and were numbered p1-p6; and six ALV-J- and MDV- or REV-infected chickens formed group P2 and were numbered p7-p12. Chickens infected with ALV-J and MDV- or REV were diagnosed by nucleic acid detection and serological examination at the College of Veterinary Medicine, Henan Agricultural University [54–56]. After being selected, each of the chickens was isolated in a cage under the same environmental conditions for 2 weeks and return to the farmer after the experiment.

All chickens were immunized against common diseases such as MD, infectious bursa disease, Newcastle disease and infectious bronchitis in accordance with established immunization procedures. The MDV vaccine was CVI988 liquid nitrogen vaccine, which was inoculated at the age of 1 day. The mortality rate of the chickens at 190 days of age was 5.2% (512/9847) for the entire flock. The anatomy of randomly selected dead chickens revealed the main manifestations of hemangioma (26/32), liver tumor (9/32), spleen tumor (5/32), proventriculus tumor (3/32) and other tumors (5/32). Cases presented alone and in combination accounted for almost 100% of the deaths.

### DNA extraction

All fecal samples were collected from the cloaca using sterile cotton swabs every 2 h from 6:00 a.m. to 18:00 p.m. and immediately placed into sterile conical tubes, frozen in liquid nitrogen, and then stored at − 80 °C until use. Samples from each chicken were mixed before use. Microbial genomic DNA extraction was carried out with a ZR fecal DNA kit (Invitrogen, Carlsbad, CA, USA) according to the manufacturer’s instructions. The quantity and quality of extracted DNA were measured using a NanoDrop ND-1000 spectrophotometer (Thermo Fisher Scientific, Waltham, MA, USA) and agarose gel electrophoresis, respectively.

### 16S rRNA amplification and MiSeq sequencing

Polymerase chain reaction (PCR) amplification of the bacterial 16S rRNA gene V3–V4 region was performed using the forward primer 338F (5′-ACTCCTACGGGAGGCAGCA-3′) and the reverse primer 806R (5′-GGACTACHVGGGTWTCTAAT-3′). Sample-specific 7-bp barcodes were incorporated into the primers for multiplex sequencing. The PCR components included 5 μl of Q5 reaction buffer (5×), 5 μl of Q5 High-Fidelity GC buffer (5×), 0.25 μl of Q5 High-Fidelity DNA Polymerase (5 U/μl), 2 μl (2.5 mM) of dNTPs, 1 μl (10 μM) of each forward and reverse primer, 2 μl of DNA template, and 8.75 μl of ddH_2_O. Thermal cycling consisted of initial denaturation at 98 °C for 2 min, followed by 25 cycles of denaturation at 98 °C for 15 s, annealing at 55 °C for 30 s, and extension at 72 °C for 30 s, and a final extension of 5 min at 72 °C. PCR amplicons were purified with Agencourt AMPure Beads (Beckman Coulter, Indianapolis, IN, USA) and quantified using a PicoGreen dsDNA Assay Kit (Invitrogen, Carlsbad, CA, USA). After the individual quantification step, amplicons were pooled in equal amounts, and paired-end 2 × 300 bp sequencing was performed using the Illumina MiSeq platform (San Diego, CA, US) with a MiSeq Reagent Kit v3 (San Diego, CA, US) at Shanghai Personal Biotechnology Co. Ltd. (Shanghai, China).

### Sequence analysis and bioinformatic analysis

The quantitative insights into microbial ecology (QIIME, v1.8.0) pipeline was employed to process the sequencing data, as previously described [57]. Low-quality sequences were filtered out through the following criteria [58, 59]: sequences with length of < 150 bp, average Phred scores of < 20, ambiguous bases, and mononucleotide repeats of > 8 bp. Paired-end reads were assembled using FLASH (v 1.2.7, http://ccb.jhu.edu/software/FLASH/) [60]. The remaining high-quality sequences were clustered into operational taxonomic units (OTUs) at 97% sequence identity by UCLUST (v0.2.0) of QIIME, and OTUs with abundance less than 0.001% of the total sequences were discarded [61]. The taxonomic information for the representative sequence in each OTU was obtained by matching the sequence database using BLAST of QIIME (v1.8.0).

Alpha-diversity indices (i.e., Chao1 estimator and Shannon estimator) were calculated using mothur (v 1.31.2). Analyses of unique OTUs and OTUs shared between the four species were conducted based on an OTU table generated by QIIME (v1.9.0). Beta diversity for both weighted and unweighted UniFrac was calculated by QIIME software (v1.9.0). Cluster analysis was preceded by principal component analysis (PCA), which was applied to reduce the dimension of the original variables using the FactoMineR package and ggplot2 package in R software (v 2.15.3). Principal coordinate analysis (PCoA) was performed to obtain principal coordinates and visualize complex, multidimensional data. PCoA results were displayed by the WGCNA package, stat packages and ggplot2 package in R software (v 2.15.3). Unweighted pair-group method with arithmetic means (UPGMA) clustering was performed as a hierarchical clustering method to interpret the distance matrix using average linkages and was conducted by QIIME software (v1.8.0). The significance of microbiota structure differentiation among groups was assessed by permutational multivariate analysis of variance (PERMANOVA) and analysis of similarities (ANOSIM) using the R package vegan (v2.5–6) [62]. Tax4Fun [63, 64] software was used to conduct microbial function prediction based on the nearest-neighbor method and the minimum 16S rRNA sequence similarity to obtain functional annotation information.

## Data Availability

The datasets used and analyzed during the current study are available from the corresponding author upon reasonable request.

## Abbreviations

ALV-J: Avian leukosis virus J
ALV-K: Avian leukosis virus K
ANOSIM: Analysis of similarities
FMT: Fecal microbiota transplantation
GALT: Gut-associated lymphoid tissue
GIT: Gastrointestinal tract
IBDV: Infectious bursal disease virus
MDV: Marek’s disease virus
MD: Marek’s disease
REV: Avian reticuloendotheliosis virus
OTU: Operational taxonomic unit
PCoA: Principal coordinate analysis
PCA: Principal component analysis
PERMANOVA: Permutational multivariate analysis of variance
